# North Dakota State Board of Veterinary Medical Examiners

**Published:** 1901-11

**Authors:** 


### NORTH DAKOTA STATE BOARD OF VETERINARY MEDICAL EXAMINERS.

THERE has been no change in the law in this State or any modification of the rules regulating its enforcement the present year. No applicants have come before the Board and no licenses have been granted. Dr. E. J. Davidson, of Grand Forks, N. D., still continues as President.

---

